# Pharmacokinetics, bioavailability, and tissue distribution of MRTX1133 in rats using UHPLC-MS/MS

**DOI:** 10.3389/fphar.2024.1509319

**Published:** 2024-12-19

**Authors:** Wei Lu, Rong Zeng, Meng Pan, Yuan Zhou, Huijuan Tang, Wanying Shen, Yijun Tang, Pan Lei

**Affiliations:** ^1^ Department of Pharmacy, Taihe Hospital, Hubei University of Medicine, Shiyan, China; ^2^ College of Basic Medical Sciences, Hubei University of Chinese Medicine, Wuhan, China; ^3^ Department of Health Management, Renmin Hospital of Wuhan University, Wuhan, China; ^4^ Department of Cardiovascular Medicine, Taihe Hospital, Hubei University of Medicine, Shiyan, China; ^5^ Hubei Hongshan Laboratory, College of Biomedicine and Health, College of Life Science and Technology, Huazhong Agricultural University, Wuhan, China; ^6^ Hubei Key Laboratory of Embryonic Stem Cell Research, Hubei Provincial Clinical Research Center for Umbilical Cord Blood Hematopoietic Stem Cells, Taihe Hospital, Hubei University of Medicine, Shiyan, China

**Keywords:** MRTX1133, UHPLC-MS/MS, pharmacokinetics, distribution, excretion

## Abstract

**Introduction:**

MRTX1133 is a selective and reversible small molecule inhibitor of KRAS (G12D), which significantly delays the progression of solid tumors. However, no study on the absorption, distribution, and excretion of MRTX1133.

**Methods:**

A fast ultra-high performance liquid chromatography-tandem quadrupole mass spectrometry method was developed for the determination of MRTX1133 in rat plasma, tissue homogenate, and urine. The method applied to the pharmacokinetics, bioavailability, tissue distribution, and excretion of MRTX1133 after oral administration (25 mg/kg) and intravenous administration (5 mg/kg).

**Results:**

The calibration curve for MRTX1133 in plasma and other homogenates was linear, with *r* ^2^ > 0.99. The intra- and inter-day accuracies were ranged from 85% to 115% and precision were within ± 10%. The matrix effect and recovery were within ± 15 %. The Cmax of MRTX1133 was 129.90 ± 25.23 ng/mL at 45 min after oral administration. The plasma half-life (t_1/2_) of MRTX1133 was 1.12 ± 0.46 h after oral administration and 2.88 ± 1.08 after intravenous administration. Its bioavailability was 2.92%. Furthermore, MRTX1133 was widely distributed in all the main organs, including liver, kidney, lung, spleen, heart, pancreas, and intestine. MRTX1133 was still detectable in liver, kidney, lung, spleen, heart, and pancreas after 24 h. The excretion ratio of prototype MRTX1133 through kidney was 22.59% ± 3.22% after 24 h.

**Conclusions:**

MRTX1133 was quickly absorbed, and widely distributed in the main organs. This study provided a reference for the quantitative determination of MTRX1133 in preclinical or clinical trials.

## Graphical Abstract

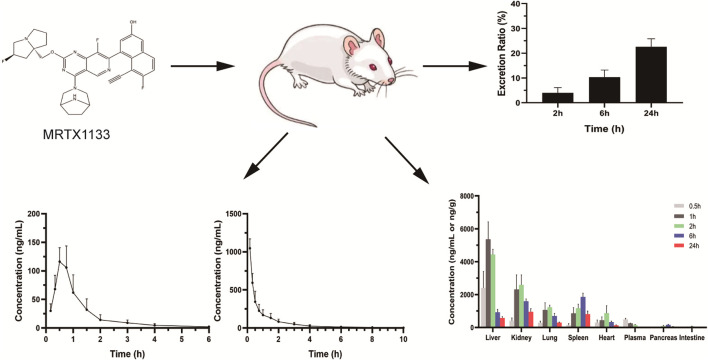

## 1 Introduction

The rat sarcoma (RAS) gene family includes Kirsten RAS (KRAS), Harvey RAS (HRAS), and neuroblastoma RAS (NRAS), which are the most prevalent and foremost genetic alteration in human cancers (Abankwa et al., 2010; Thein et al., 2021). KRAS is the most frequently mutated among the three isoforms; more than 85% of all cancers carry KRAS aberrations. The highest rate of KRAS mutations is present in pancreatic (∼90%) and colorectal cancer (30–40%), as well as lung adenocarcinoma (∼32%) (Simanshu et al., 2017). The 80% of carcinogenic mutations in KRAS mutated tumors occur in the codon 12, and the most popular mutation sites are KRAS (G12D), KRAS (G12V) and KRAS (G12C) (Prior et al., 2012).

As the most frequently mutated isoform, KRAS has been extensively studied in the past years (Liu et al., 2019). Many small molecular compounds have been designed to inhibit KRAS mutations, which target upstream regulators, downstream effectors, and the mutant KRAS protein itself (Bannoura et al., 2021; Tang et al., 2021; Qian et al., 2020; Mao et al., 2022). However, their poor metabolic stability and/or bioavailability affect KRAS drug ability. Only Sotorasib (AMG 510) (Canon et al., 2019) and Adagrasib (MRTX849) (Hallin et al., 2020) have been recently approved in the treatment of KRAS (G12C)-mutated locally advanced or metastatic non-small-cell lung cancer (Reck et al., 2021). The prevalence of the G12D oncogenic mutation is much higher than others including G12C in pancreatic cancer and lung cancer (Prior et al., 2012; Ryan and Corcoran, 2018), but no inhibitor has been approved for the KRAS (G12D)-mutated cancer.

MRTX1133 is the first small molecule inhibitor of KRAS (G12D) reported in the literature that exerts a robust *in vivo* efficacy, and its antitumor effect was demonstrated in a murine animal model (Wang et al., 2022). MRTX1133 is selective and a reversible inhibitor of KRAS (G12D), and it binds to and inhibits mutant KRAS protein in both its active and inactive states. MRTX1133 exhibits single digit nanomolar potency and is more than 1000-fold selective for KRAS (G12D) compared with wild-type KRAS, as revealed *in vitro* in cell culture. MRTX1133 induces tumor regression in multiple *in vivo* tumor models, including pancreatic and colorectal cancer (Kataoka et al., 2023; Drosten and Barbacid, 2022).

However, no analytical liquid chromatography tandem mass spectrometry (LC-MS) method or pharmacokinetics are reported for MRTX1133 in plasma. The quantification of plasma concentration and the comprehensive understanding of the pharmacokinetics are indispensable for a better medication and further benefit for patients. Therefore, a reproducible UHPLC-MS/MS assay was developed and fully validated to determine the concentration of MRTX1133 in rats’ plasma. Furthermore, the method was successfully used in a pharmacokinetic study on rats after oral and intravenous administration to evaluate tissue distribution and drug excretion. The results might provide a significant basis for future KRAS targeted drug development, pharmacokinetic investigation and therapeutic drug monitoring in preclinical or clinical studies/trials.

## 2 Materials and methods

### 2.1 Chemicals and reagents

MRTX1133 (purity >98%) was purchased from Shanghai Ronbio Scientific Co., Ltd. (Shanghai, China) and tinidazole (purity = 99.85%) was purchased from the National Institutes for Food and Drug Control (Beijing, China). Formic acid was purchased from Tianjin Kemiou Chemical Reagent Co., Ltd (Tianjin, China). Redistilled and deionized water was used overall the entire the study. HPLC grade acetonitrile and methanol were purchased from Merck (Darmstadt, Germany).

### 2.2 Instrumentation and LC-MS/MS conditions

**FIGURE 1:**
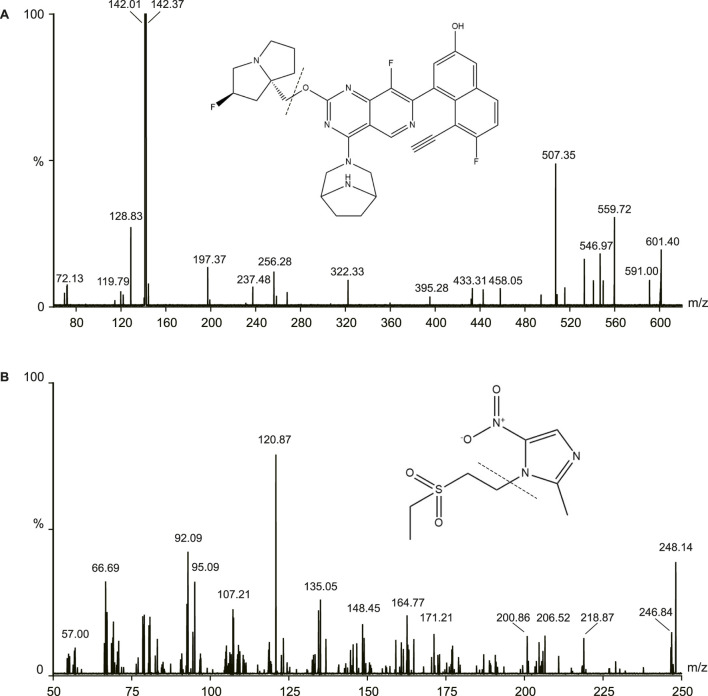
Mass fragmentation pattern of MRTX1133 **(A)** and tinidazole **(B)**.

The samples were measured using Waters ACQUITY UHPLC system (Waters, Milford, MA, United States) and a Micro mass Quattro Micro API mass spectrometer (Waters, Milford, MA, United States) in this study. The electrospray ionization source operated in the positive ionization mode. The following parameters were used: capillary voltage: 3.0 kV, source temperature: 150°C, desolvation temperature was 400°C. The MS/MS parameters were 601.1/142.4 for MRTX1133 and 248.1/120.9 for tinidazole. The cone energies were 26V (MRTX1133) and 30V (tinidazole), the collision energies were 22V (MRTX1133) and 16V (tinidazole). The fragmentation pattern for MRTX1133 and tinidazole in positive ion mode is shown in Figure 1.

Chromatographic separation was performed on an ACQUITY™ UHPLC BEH C_18_ column (2.1 × 50 mm; 1.7 µm). The mobile phases were 0.1% formic acid in water (solvent A) and acetonitrile (solvent B), the flow rate was 0.3 mL min^-1^. The fast gradient elution program was the following: 0–0.5 min, 10% B; 0.5–3.0 min, 10%–90% B; 3.0–4.0 min, 90% B; 4.0–4.5 min, 90%–10% B; 4.5–5.0 min, 10% B. The column was maintained at 40°C, and the injection volume was 5 µL.

### 2.3 Preparation of standard and quality control (QC) samples

The stock solution of MRTX1133 was at the concentration of 13.2 mg/mL in methanol. The working solutions of MRTX1133 were obtained by diluting the stock solution in methanol until reaching the following concentrations: 1.32, 2.64, 6.60, 13.20, 26.40, 66.00, 132.00, 264.00, 660.00, 1,320.00 and 2,640.00 ng mL^-1^. The QC solutions of MRTX1133 were prepared by diluting the stock solution in methanol until reaching the concentrations of 2,112.00 ng mL^-1^, 1,056.00 ng mL^-1^, 211.20 ng mL^-1^, 105.60 ng mL^-1^, 21.12 ng mL^-1^, and 4.22 ng mL^-1^. A stock solution of tinidazole was prepared in methanol at 10.32 mg mL^-1^, then it was diluted in methanol to the working solution of 20.64 ng mL^-1^. All stock and working solutions were stored at 4°C.

The calibration solution and QC solution of MRTX1133 (100 µL) were added to centrifuge tubes and evaporated under nitrogen. Next, 100 µL blank plasma and tissue homogenate were added and mixed by vortexing for 5 min to prepare the samples of calibration curve, plasma QC samples and homogenate QC samples. The calibration curve samples, and QC samples were treated by the protocol described in the sample preparation. All calibration samples were freshly prepared before analysis.

### 2.4 Sample preparation

100 µL sample plasma and 100 µL tinidazole solution (20.64 ng mL^-1^) were mixed, and 500 µL methanol were added next. The mixture was vortexed for 60 s and centrifugated for 10 min (20,800 × *g* at 4°C). The supernatant was transferred to a new tube and evaporated to dryness under nitrogen. The obtained residue was reconstituted in 100 mL 10% acetonitrile - water (containing 0.1% formic acid) and centrifuged (20,800 × *g* for 10 min, 4°C). Then, 5 µL supernatant were injected into the UHPLC-MS/MS system for analysis.

### 2.5 Method validation

The method validation of MRTX1133 was referred to the principles of the bioanalytical method validation guidelines (U.S. Department of Health and Human Services et al., 2018), and it was including selectivity, specificity, calibration curve, matrix effects, extraction recovery, precision and accuracy, stability.

#### 2.5.1 Selectivity, specificity, and carry-over

The selectivity and specificity of the method were evaluated by comparing the chromatograms of different sources blank rat plasma, blank rat plasma spiked with MRTX1133 at LLOQ, and the plasma samples after oral administration of MRTX1133. Carry-over was assessed by comparing an extract of blank plasma injected immediately after the highest calibration standard injected in triplicate.

#### 2.5.2 Linearity of the calibration curve and LLOQ

Linearity was evaluated by analyzing the calibration curve using 7 concentrations. The calibration curve was constructed by plotting peak area ratios (analyte/internal standard) *versus* plasma concentrations. Linear weighted least-squares analysis was performed, and a weighting factor of 1/x^2^ was used. A coefficient of determination *r* ^2^ > 0.99 was expected in all calibration curves.

#### 2.5.3 Matrix effects and extraction recovery

The extraction recovery of MRTX1133 was assessed by comparing the peak area at LLOQ, LQ, MQ, and HQ that were spiked with analytes prior to extraction with the peak area of those that were spiked with blank biological samples. The matrix effects were evaluated by comparing the peak areas of the analytes in post-extracted blank biological samples spiked with QC samples with those of pure standard solutions with the same concentration that were dried directly and reconstituted with the mobile phase. All matrix effects and extraction recoveries were determined at four concentrations, and the QC samples were prepared using one source of plasma. The ratio of extraction recoveries should be >85% and <115%, while the relative standard deviation (RSD) should be <15%.

#### 2.5.4 Precision and accuracy

Intra-day precision and accuracy were evaluated in six replicates at LLOQ, LQ, MQ, and HQ within 1 day. Inter-day precision and accuracy were assessed according to the analysis of the same QC samples on three consecutive days. RSD was evaluated to determine precision, and accuracy was characterized as the extraction recovery. The RSD should be within 15%, and the accuracy should be in 80%–120%.

#### 2.5.5 Stability

The stability of MRTX1133 was determined by analyzing LQ, MQ, and HQ samples under different storage conditions, which included three freeze-thaw stability (from −20°C to 25°C), long-term stability (−80°C for 14 days), short-term stability (25°C for 6 h), and post-processing stability (4°C for 24 h). The samples concentrations were compared with the freshly prepared QC samples and were considered stable if the accuracy were 85%–115% and RSD were ±15%.

### 2.6 Animal study

45 Male Sprague-Dawley rats (180–220 g) were purchased from Hubei University of Medicine Animal Laboratory (Shiyan, China). The rats were fasted overnight before the day of the experiment. The animal study protocols were approved by Hubei University of Medicine’s Institutional Animal Care and Uses Committee, and all the animal-related experimental procedures were conducted in accordance with the ARRIVE guidelines.

The pharmacokinetic study was performed using oral solutions of MRTX1133 in 5% carboxymethyl-cellulose sodium (CMC-Na) at a concentration of 2.5 mg mL^-1^ (the dose was 25 mg kg^-1^ and 10 mL kg^-1^). The intravenous solution was prepared at a concentration of 5 mg mL^-1^ in polyethylene glycol 400 and dimethyl sulfoxide (8%). Twenty rats were randomly divided into two groups (ten rats each group), the rats in one group were given MRTX1133 by intravenous administration at 5 mg kg^-1^ and the other group by intragastric administration at 25 mg kg^-1^. Rats were slightly anesthetized with diethyl ether, blood samples were collected (approximately 150 µL) from the suborbital vein, and placed into heparinized tubes at 0.17 h, 0.33 h, 0.5h, 0.75 h, 1 h, 1.5 h, 2 h, 3 h, 4 h, 6 h, 8 h, 10 h, and 24 h after treatment. The rats were treated with saline at the same volume by gavage and used as control. Blood samples were centrifuged at 20,800 *g* for 10 min (at 4°C) and stored at - 80°C.

The distribution of MRTX1133 in the tissues was performed using 25 rats with a single intravenous administration of MRTX1133 at 5 mg kg^-1^. The blood samples were collected from abdominal aorta at 0.5 h, 1 h, 2 h, 6 h, and 24 h (five rats at each time point), then the organs such as the heart, liver, spleen, lung, kidney, intestine, and pancreas were immediately collected. The organs were washed with cold physiological saline (4°C), they were weighed and homogenized in cold saline solution (1:3, w/v). The homogenized samples were treated according to the method described in the sample preparation section. The excretion of MRTX1133 in the urine was also investigated by placing the rats in metabolic cages after intravenous administration of MRTX1133 at 5 mg kg^-1^ (one rat in each cage), the urine and feces were collected at 2 h, 6 h and 24 h after administration (five rats at each time point), and the content of MRTX1133 in urine and feces was detected by the LC-MS method.

The DAS 3.2 software package (edited by the Chinese Mathematical Pharmacology Society) was used for the pharmacokinetic data analysis and the non-compartmental model was applied. The oral bioavailability (*F*) of MRTX1133 was measured by comparing each area under the curve (AUC) 0-t value after intragastric (i.g.) and intravenous (i.v.) administration according to the equation: *F* = (AUC i. g./Dose i. g.)/(AUC i. v./Dose i. v.).

## 3 Results and discussion

### 3.1 Method validation

#### 3.1.1 Selectivity, specificity, and carry-over

**FIGURE 2:**
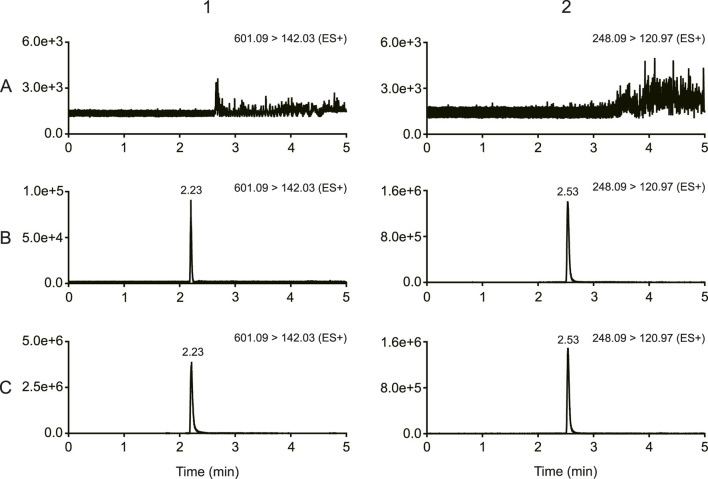
Typical chromatograms of MRTX1133 and tinidazole **(A)** Blank rat plasma sample; **(B)** blank rat plasma sample spiked with MRTX1133 at LLOQ and tinidazole (20.64 ng/mL); **(C)** rat plasma sample at 30 min after intravenous injection of 5 mg/kg MRTX1133 spiked with tinidazole (20.64 ng/mL). The number 1 is MRTX1133, 2 is tinidazole.

As shown in Figure 2, there was no obvious interferences in the samples. The peak area of MRTX1133 and tinidazole in the blank plasma sample and tissue homogenate injected after the higher limit of quantification sample was <3% for MRTX1133 and <0.8% for tinidazole. The results show that the LC-MS/MS method had good selectivity and specificity in plasma and tissue homogenate, and the carry-over effect conforms the requirement.

#### 3.1.2 Linearity and LLOQ

The calibration curve and linear range in different samples were shown in Table 1. The result shown that the calibration curves fitted well (*r* ^2^ > 0.99). All the LLOQ were 1.32 ng/mL, and the S/N ratio of LLOQ was higher than >10.

**TABLE 1:** Results of linearity relations and LLOQ.

Samples	Calibration equations	Standard curve (ng/mL)	*r* ^*2*^
Plasma	Y = 0.0143 X + 0.0019	1.32–1,320.00	0.9998
Heart	Y = 0.0445 X - 0.0360	13.20–1,320.00	0.9996
Liver	Y = 0.0319 X + 0.0457	13.20–2,640.00	0.9992
Lung	Y = 0.0471 X + 0.0114	13.20–1,320.00	0.9994
Kidney	Y = 0.0467 X + 0.0175	13.20–1,320.00	0.9988
Spleen	Y = 0.0480 X + 0.0570	13.20–1,320.00	0.9996
Pancreas	Y = 0.0506 X + 0.0354	1.32–132.00	0.9991
Intestine	Y = 0.0518 X + 0.0438	1.32–132.00	0.9990

#### 3.1.3 Precision and accuracy

The accuracy and intra- and inter-day precisions are shown in Table 2. The accuracy of four levels of QCs samples ranged from 88.07% to 110.17% in plasma and tissues homogenate. The intra- and inter-day precision of the MRTX1133 ranged from 1.31% to 8.16%. The results demonstrated that the method was reliable and reproducible.

**TABLE 2:** Intra-and inter-day accuracy and precision of MTXR1133 in rat plasma and tissues homogenate.

Samples	Nominal concentration ng/mL	Intra-day (%, n = 6)		Inter-day (%, n = 18)	
		Accuracy	RSD	Accuracy	RSD
Plasma	1.32	110.17	3.95	108.34	6.70
	4.22	108.24	1.31	105.99	3.37
	105.60	95.23	4.86	93.11	4.00
	1,056.00	93.09	3.59	96.43	5.59
Heart	13.20	106.56	2.55	106.47	2.79
	21.12	103.53	4.40	102.84	4.20
	211.20	98.33	4.46	97.06	3.37
	1,056.00	95.63	1.83	94.57	2.31
Liver	13.20	105.29	5.01	97.48	4.56
	21.12	104.43	3.12	102.06	8.16
	211.20	103.27	3.04	100.81	8.04
	2,112.00	97.90	5.24	99.15	6.57
Lung	13.20	102.98	4.36	105.28	3.73
	21.12	96.49	2.41	102.85	4.2
	211.20	94.67	2.36	97.06	3.37
	1,056.00	93.05	2.33	94.57	2.31
Kidney	13.20	105.33	2.99	102.59	4.09
	21.12	103.09	4.21	97.87	6.71
	211.20	101.15	2.61	96.77	6.08
	1,056.00	94.86	2.86	94.56	3.41
Spleen	13.20	102.28	2.68	102.16	7.64
	21.12	95.36	2.53	95.5	3.21
	211.20	94.06	5.88	95.16	4.21
	1,056.00	91.76	2.29	91.64	2.46
Pancreas	1.32	88.06	4.4	88.65	5.26
	4.22	97.18	4.26	93.4	5.55
	21.12	104.10	1.79	102.45	3.62
	105.60	96.87	2.46	93.96	4.14
Intestine	1.32	89.83	5.07	88.6	5.43
	4.22	98.3	4.92	94.85	5.02
	21.12	104.1	1.80	102.45	3.62
	105.60	100.12	2.27	95.74	5.32

#### 3.1.4 Recovery and matrix effect

As shown in Table 3, the extraction recovery was ranged from 90.63% to 104.91% in plasma and homogenates, the matrix effect was ranged 95.25% and 105.22% in plasma and homogenates. All the RSD was <9.78%. The result demonstrated that the sample pretreatment could be used to obtain a stable extraction efficiency, and the matrix could be ignored.

**TABLE 3:** Matrix effect and extraction recovery of MTXR1133 in rat plasma and homogenate (%, n = 6).

Samples	Nominal concentration ng/mL	Extraction recovery		Matrix effect	
		Accuracy	RSD	Accuracy	RSD
Plasma	1.32	90.63	3.59	100.17	7.70
	4.22	95.92	1.71	99.40	4.80
	105.60	103.19	4.96	96.72	2.90
	1,056.00	104.66	4.05	103.20	4.24
Heart	13.20	98.07	3.82	96.59	2.86
	21.12	94.75	4.23	95.86	4.67
	211.20	98.46	5.48	97.96	5.05
	1,056.00	101.94	2.72	95.75	3.33
Liver	13.20	93.67	7.31	96.46	5.52
	21.12	98.99	4.10	101.71	5.94
	211.20	95.97	3.78	101.46	5.79
	2,112.00	94.96	5.15	99.43	3.36
Lung	13.20	104.91	6.30	98.94	3.22
	21.12	96.98	2.31	95.24	3.87
	211.20	96.72	2.91	97.74	4.30
	1,056.00	97.49	3.23	104.50	4.00
Kidney	13.20	97.22	4.69	97.35	4.51
	21.12	96.45	7.43	99.65	3.60
	211.20	95.22	3.58	104.02	3.19
	1,056.00	93.33	4.64	103.22	2.32
Spleen	13.20	95.78	2.91	104.37	3.98
	21.12	92.33	5.43	101.94	5.49
	211.20	92.08	8.77	95.42	1.97
	1,056.00	96.84	3.02	101.08	4.46
Pancreas	1.32	92.50	5.94	102.16	6.06
	4.22	90.63	5.92	106.82	3.73
	21.12	93.57	5.09	102.97	5.45
	105.60	91.60	4.81	98.57	2.75
Intestine	1.32	93.45	7.34	102.02	4.59
	4.22	91.62	5.98	104.46	3.26
	21.12	92.86	1.98	98.98	3.94
	105.60	93.12	2.36	100.25	4.72

#### 3.1.5 Stability

The stability of the method is shown in Table 4. MRTX1133 was stable in plasma samples and homogenates stored at 25°C for 6 h, after storage in the sample manager at 4°C for 24 h, after three freeze-thaw cycles (from −20°C to 25°C) and at −80°C for 14 days. The accuracy was between 90.04% and 108.92% in plasma, liver homogenate, kidney homogenate, and intestine homogenate. All the RSD were within 9.57%.

**TABLE 4:** Stability of MRTX1133 in rat plasma at different storage conditions (%, n = 6).

Samples	Nominal concentration (ng/mL)	Short-term stability		Post-processing stability		Freeze-thaw stability		−80°C for 14 days	
		Accuracy	RSD	Accuracy	RSD	Accuracy	RSD	Accuracy	RSD
Plasma	4.22	108.92	2.56	107.16	1.05	101.64	4.54	103.66	3.09
	105.60	95.35	1.98	94.98	5.20	92.05	2.07	90.18	1.86
	1,056.00	92.49	3.61	92.05	1.82	90.04	1.55	90.23	3.00
Liver	21.12	101.44	3.51	104.23	3.91	97.72	5.93	96.4	4.36
	211.20	105.89	3.82	105.35	2.29	96.69	2.41	94.88	1.70
	2,112.00	98.17	4.03	97.47	2.45	92.38	3.15	93.33	2.16
Kidney	21.12	103.01	5.43	101.18	3.68	95.07	2.28	95.23	2.94
	211.20	97.29	3.39	98.13	3.13	95.58	2.12	92.7	3.69
	1,056.00	97.04	2.13	95.47	2.10	93.72	3.72	91.34	2.97
Intestine	4.22	95.41	6.34	98.23	5.69	95.83	5.04	93.31	5.85
	21.12	96.69	5.91	98.14	6.08	94.42	5.50	92.54	3.00
	105.60	94.97	3.17	90.73	3.34	90.73	3.34	90.35	3.25

### 3.2 Animal study

#### 3.2.1 Pharmacokinetics study

**FIGURE 3:**
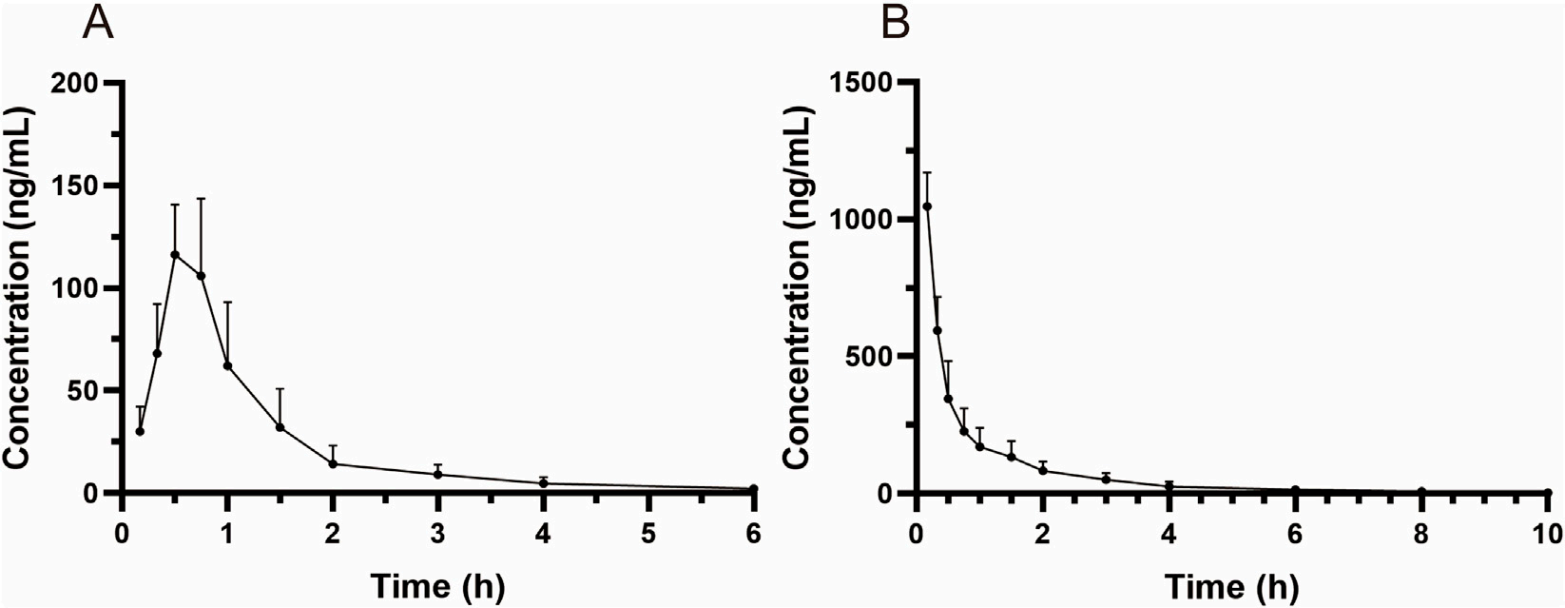
Mean plasma concentration-time curves in rats after intragastric administration (25 mg/kg) **(A)** and intravenous administration (5 mg/kg) **(B)**. Values are expressed as mean ± SD (n = 10).

MRTX1133 was detected in rat plasma samples after tail vein injection of 5 mg kg^-1^ and intragastric administration of 25 mg kg^-1^. The plasma concentration-time profile of MRTX1133 is shown in Figure 3, and the pharmacokinetic parameters are summarized in Table 5. The plasma concentration of MRTX1133 sharply increased after the oral administration, reaching the peak concentration at 45 min with the plasma C_max_ of 129.90 ± 25.23 ng mL^-1^, suggesting that MRTX1133 was quickly absorbed. The t_1/2_ of MRTX1133 was 1.12 ± 0.46 h after oral administration and 2.88 ± 1.08 h after intravenous administration. MRTX1133 was bleow the limit of quantitation 6 h after oral administration and 8 h after intravenous administration. Wang et al. (2022) investigated the concentration of MRTX1133 in CD-1 mice after intraperitoneal administration of 30 mg kg^-1^. Their result showed that the C _max_ of MRTX1133 was approximately 7,000 ng mL^-1^ at 1 h after intraperitoneal administration, almost 1,000 ng mL^-1^ at 4 h, 100 ng mL^-1^ at 8 h, and it was detected up to 24 h (approximately 50 ng mL^-1^), indicating that MRTX1133 has a long plasma half-life time, our results were quite different than those. The reasons of the inconsistent results might be different animals and administration routes. The AUC values for the oral and intravenous administration of MRTX1133 were 135.54 ± 46.51 and 927.88 ± 192.11 μg/L*h; thus, the *F* of MRTX1133 in rats was 2.92%. Suggesting that MRTX1133 had a very low bioavailability. The low bioavailability could be caused by different factors; therefore, it needed to be further studied in the process of formulation development.

**TABLE 5:** Pharmacokinetic parameters of MRTX1133 in rat plasma after intragastric administration (25 mg kg^-1^) and intravenous injection (5 mg kg^-1^).

Parameters	Oral	Intravenous
AUC_(0-t)_/µg/L*h	135.54 ± 46.51	927.88 ± 192.11
MRT_(0-t)_/h	1.25 ± 0.18	1.34 ± 0.60
C_max_/ng·mL^-1^	129.90 ± 25.23	
T_max_/h	0.60 ± 0.13	
*t*_*1/2*_/h	1.12 ± 0.46	2.88 ± 1.08
V/F/L·kg^-1^	318.99 ± 157.43	22.83 ± 11.67
CL/F/L/h/kg	199.30 ± 61.48	5.14 ± 1.58
*F*/%	2.92	

#### 3.2.2 Tissue distribution and excretion study

**FIGURE 4:**
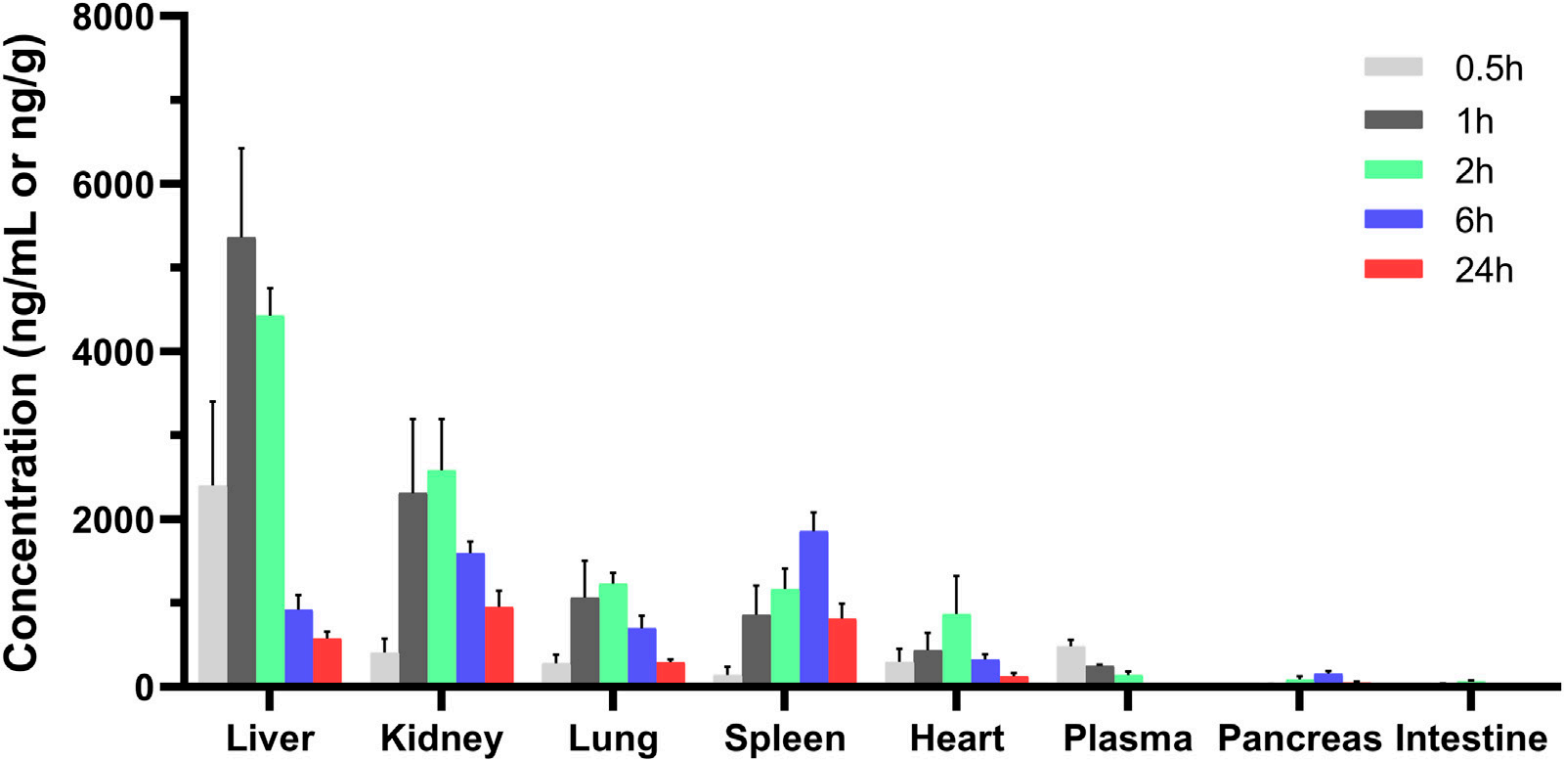
Mean concentration - time profiles of MRTX1133 in plasma (ng/mL), liver, heart, spleen, lung, kidney, pancreas, and intestine (ng/g) after intravenous administration of MRTX1133 at different times at a dose of 5 mg/kg in rat. Values are expressed as mean ± SD (n = 5).

The tissue distribution of MRTX1133 was shown in Figure 4. MRTX1133 was widely distributed in the main organs, such as liver, kidney, lung, spleen, heart, pancreas, and intestine. The highest concentrations of MRTX1133 in the liver was 5,358.68 ± 1,062.23 ng g^-1^ at 1 h after administration, the highest concentrations in the kidney, lung and heart were 2,584.60 ± 609.56 ng g^-1^, 1,230.62 ± 125.94 ng g^-1^ and 879.29 ± 449.87 ng g^-1^, respectively, after 2 h. The highest concentrations in the spleen and pancreas were 1858.73 ± 224.31 ng g^-1^ and 155.74 ± 34.18 ng g^-1^, respectively, after 6 h. MRTX1133 was still detectable in the organs (except intestine) at 24 h after administration. The concentration of MRTX1133 in liver, spleen, kidney, and lung increased rapidly after administration, and they were higher than the concentration of the drug in plasma at 2 h after administration, which is indicated that MRTX1133 has high affinity to these organs. MRTX1133 was quickly transferred from serum to the liver, sleep, lung, and kidney, and it was widely distributed into the tissues. The testis was also assessed, but the results showed no MRTX1133.

**FIGURE 5:**
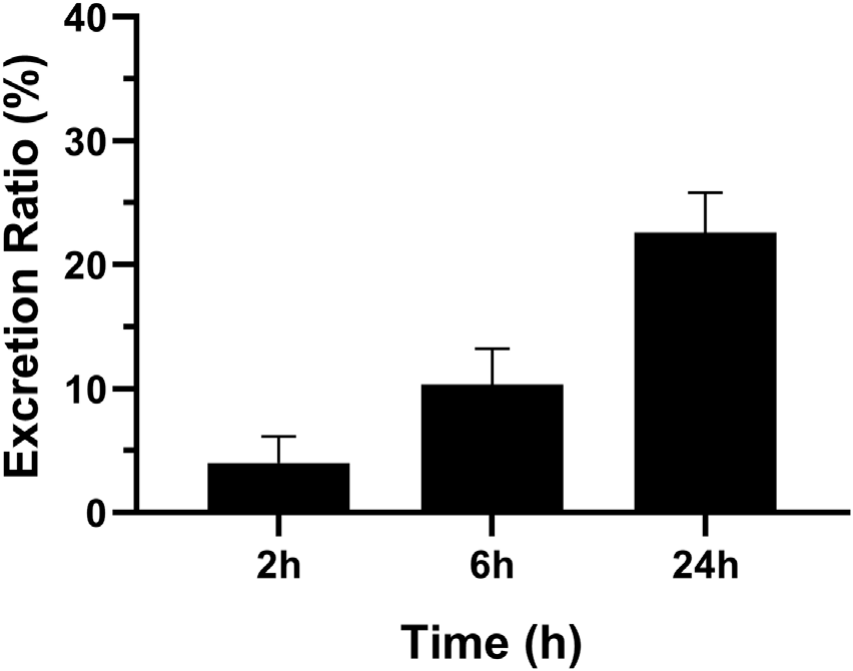
Mean excretion ratio of MRTX1133 in rat urine (%) at 2 h, 6 h and 24 h after intravenous administration of 5 mg/kg. Values are expressed as mean ± SD (n = 5).

The concentration of MRTX1133 in urine was 10.43% ± 2.89% (6.53%–13.54%) excreted through the kidney at 6 h after administration as the prototype drug, and 22.59% ± 3.22% (17.60%–25.92%) was excreted 24 h as the prototype drug. The result is shown in Figure 5. However, no MRTX1133 was found in the feces. The results of tissue distribution and excretion study revealed that MRTX 1133 might not be excreted through the biliary route. In addition, the excretion ratio through the kidney of the prototype drugs was very low because most drug might be metabolized into other components by the liver.

This study investigated the pharmacokinetic, distribution and excretion of MRTX1133 in rats, but some limitations are present. We only collected the feces 24 h after the administration of the drug, and the feces in other time was not collected, so we could not determine whether MRTX1133 was excreted with feces. Although MRTX1133 was not detected in the feces, bile was not collected; thus, it was not possible to accurately evaluate whether MRTX1133 was excreted through the bile. In addition, the metabolic process and main products of MRTX 1133 were not investigated *in vivo*.

## 4 Conclusion

This study was the first to evaluate the pharmacokinetics, bioavailability, distribution, and excretion of MTRX1133 in rats. A sensitive, rapid, and reliable UHPLC-MS/MS method was developed to measure MTRX1133 in rat plasma, tissue homogenate and urine. The established method was successfully applied to the pharmacokinetic study of MTRX1133 in rats after administration by different routes. MRTX1133 is quickly absorbed after oral administration and widely distributed in the body. But the bioavailability is very low and only 24% of the drug were excreted through the kidneys by the original form. This study might provide a sufficient reference for the quantitative determination of MTRX1133, in preclinical or clinical studies/trials.

## Data Availability

The raw data supporting the conclusions of this article will be made available by the authors, without undue reservation.
