# Supplementary figures and images for: Pharmacokinetics, bioavailability, and tissue distribution of MRTX1133 in rats using UHPLC-MS/MS
Source: Front Pharmacol. 2024 Dec 19;15:1509319. doi: 10.3389/fphar.2024.1509319 (PMC11693508; doi:10.3389/fphar.2024.1509319)

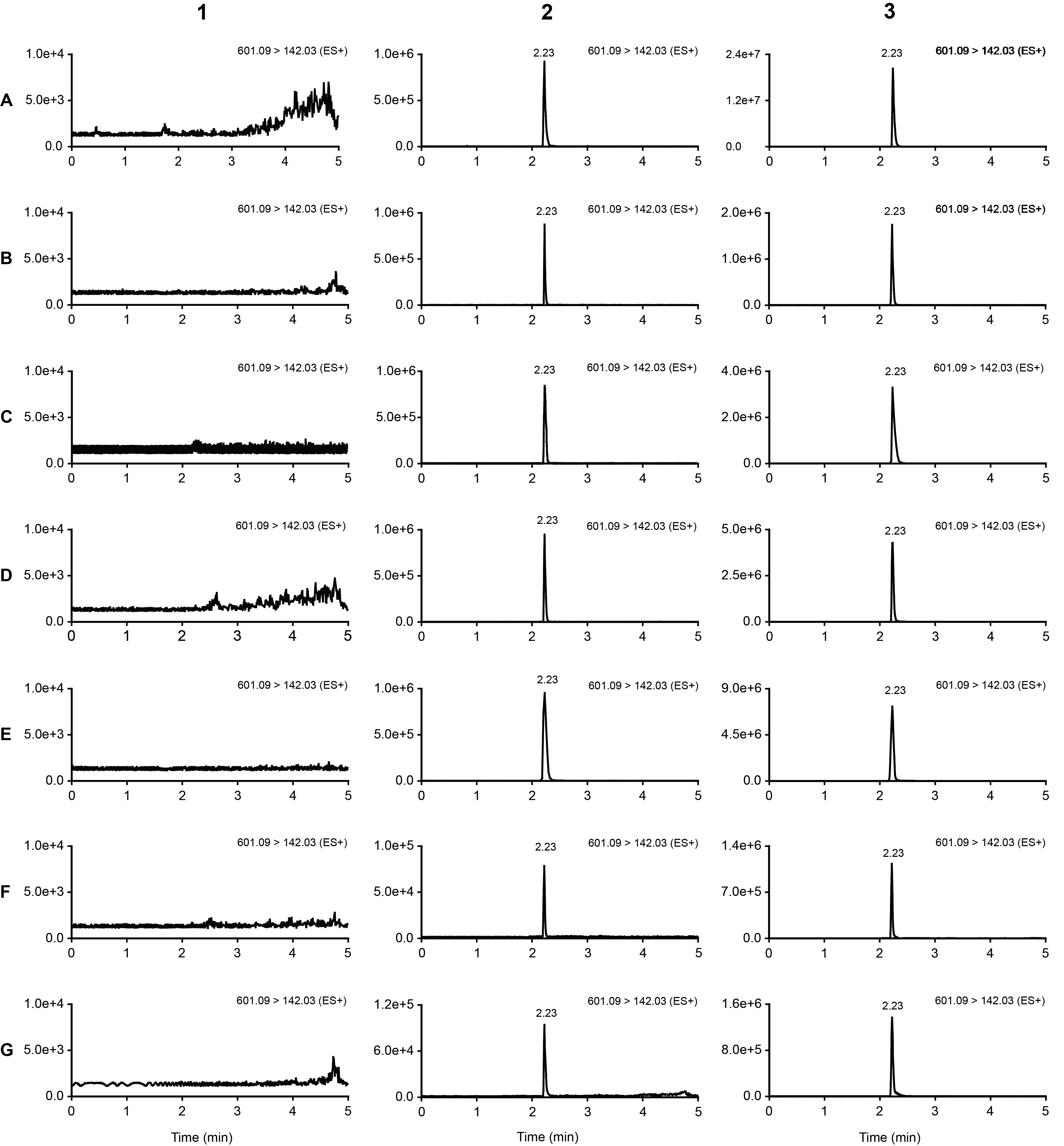

Supplement: Supplementary file 1 [file Image1.jpeg]
